# Correction to ‘A modified formulation of quasi-linear viscoelasticity for transversely isotropic materials under finite deformation’

**DOI:** 10.1098/rspa.2022.0069

**Published:** 2022-02

**Authors:** Valentina Balbi, Tom Shearer, William Parnell

*Proc. R. Soc. A* **474**, 20180231. (Published online 19 September 2018). (doi:10.1098/rspa.2018.0231)

— The vector M should not appear in equation (1.2).
— After equation (3.6), the text should be: ‘We note that the bases introduced in (A 12) and (A 13) may depend on the deformation through the normalized vector:
  $$\hat{\text{m}}(t)=\frac{\text{m}(t)}{||\text{m}(t)||}=\frac{\text{F}(t)\text{M}}{\sqrt{\text{F}(t)\text{M}\cdot \text{F}(t)\text{M}}}.$$
  However, in this paper, we focus on three deformation modes, namely uni-axial extension in the direction of the fibres, in-plane simple shear and longitudinal shear, i.e. shear in the fibre-direction. For these modes, the bases do not depend on the deformation, therefore $\hat{\text{m}}(t)=\text{M}$ and the time derivative of the tensor G in equation (3.6) only acts on the components $G_{n}$ giving rise to the constitutive equation (3.7). This modified version of the QLV theory still preserves the property of the relaxation functions being independent of the deformation. The more general case, where the bases explicitly depend on the deformation will be considered in a future paper. Finally, we note that in the small deformation limit, the two deformed fibre vectors and the undeformed vector coincide, i.e. $\hat{\text{m}}(t)=\text{m}(t)=\text{M},\forall t$.’
— In equations (3.14) and (3.15), m should be replaced by $\hat{\text{m}}$.
— In equation (4.14), ${\tilde{T}}^{e}$ should be replaced by $\Pi_{\text{L}33}^{e}$ and equation (4.15) should be $\Pi_{\text{L}33}^{e}={\tilde{T}}^{e}/\Lambda^{2}=T_{33}^{e}/\Lambda^{2}$.

— Equation (4.16) should be replaced by: $T_{33}(t)=T_{33}^{e}(t)+\Lambda^{2}(t)\int_{0}^{t}(R^{\prime }(t-\tau )/E_{L})(T_{33}^{\text{e}}(\tau )/\Lambda^{2}(\tau ))\;\text{d}\tau$.
— The caption of figure 1 should be updated as follows: ‘(a) A block of a TI material with fibres pointing in the direction of the ${\text{E}}_{3}$-axis (i.e. $\text{M}={\text{E}}_{3}$), (b) under the simple transverse shear deformation in equation (4.17) and (c) under the simple longitudinal shear deformation in equation (4.24). Here, $\left\{{\text{E}}_{1},{\text{E}}_{2},{\text{E}}_{3}\right\}$ and $\left\{{\text{e}}_{1},{\text{e}}_{2},{\text{e}}_{3}\right\}$ are the basis vector in the undeformed and the deformed configurations, respectively.’
— According to the corrected version of equation (4.16), figure 3*a* and 3*d* should be replaced by figure 1*a*,*b* below, respectively.

**Figure 1.**
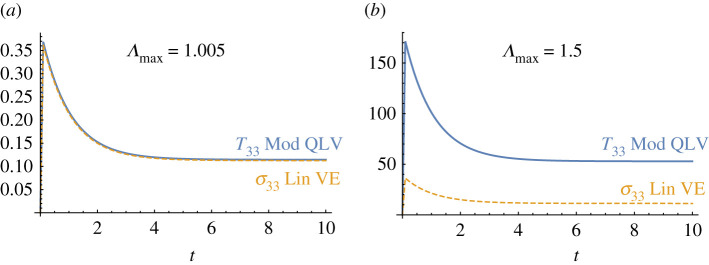
The curves are obtained by setting: $E_{L\infty }/E_{L}=0.3$, $\tau_{R}=1$, $\mu_{T\infty }/\mu_{T}=0.9$, $\tau_{5}=2$, $\mu_{L\infty }/\mu_{L}=0.8$, $\tau_{6}=1.5$, $E_{L}/\mu_{T}=75$, $\mu_{L}/\mu_{T}=5$, $\alpha_{MR}=0.25$ and the rising time of the ramp is 0.1s. All stresses shown are normalized by $\mu_{T}$.

In the appendix, the following corrections apply:

— Before (A1), the equation for Θ should be: $\Theta =\text{I}-\hat{\text{m}}⊗\hat{\text{m}}$.
— In (A1), (A2) and (A3) all m should be replaced by $\hat{m}$ and in section (c) of appendix A, all m should be replaced by $\hat{\text{m}}$.
— In (A17), the equation for $\sigma_{6}^{e}$ should be: $\sigma_{6}^{e}=\sigma_{m}^{e}-2\sigma_{||}^{e}\hat{\text{m}}⊗\hat{\text{m}}$.
— In (A18), the second equation should be ${\bar{\sigma }}^{e}=\sigma_{||}^{e}-2{\tilde{\sigma }}^{e}$.
— In (A26), **M** should be replaced by ${\text{F}}^{-1}\hat{\text{m}}$ and the last term should be deleted.

## Data accessibility

This article has no additional data.

